# C-C Chemokine Receptor 2 Inhibitor Ameliorates Hepatic Steatosis by Improving ER Stress and Inflammation in a Type 2 Diabetic Mouse Model

**DOI:** 10.1371/journal.pone.0120711

**Published:** 2015-03-27

**Authors:** Hong-Min Kim, Eun Soo Lee, Bo Ra Lee, Dhananjay Yadav, You Mi Kim, Hyun-Jeong Ko, Kyu Sang Park, Eun Young Lee, Choon Hee Chung

**Affiliations:** 1 Department of Medicine, Graduate School, Yonsei University, Wonju, Korea; 2 College of Pharmacy, Kangwon National University, Chuncheon, Korea; 3 Department of Internal Medicine, Soonchunhyang University Cheonan Hospital, Cheonan, Korea; 4 Department of Physiology, Graduate School, Yonsei University, Wonju, Korea; 5 Institute of Lifestyle Medicine, Yonsei University, Wonju, Korea; Hosptial Infantil Universitario Niño Jesús, CIBEROBN, SPAIN

## Abstract

Hepatic steatosis is the accumulation of excess fat in the liver. Recently, hepatic steatosis has become more important because it occurs in the patients with obesity, type 2 diabetes, and hyperlipidemia and is associated with endoplasmic reticulum (ER) stress and insulin resistance. C-C chemokine receptor 2 (CCR2) inhibitor has been reported to improve inflammation and glucose intolerance in diabetes, but its mechanisms remained unknown in hepatic steatosis. We examined whether CCR2 inhibitor improves ER stress-induced hepatic steatosis in type 2 diabetic mice. In this study, *db/db* and *db/m* (n = 9) mice were fed CCR2 inhibitor (2 mg/kg/day) for 9 weeks. In diabetic mice, CCR2 inhibitor decreased plasma and hepatic triglycerides levels and improved insulin sensitivity. Moreover, CCR2 inhibitor treatment decreased ER stress markers (e.g., BiP, ATF4, CHOP, and XBP-1) and inflammatory cytokines (e.g., TNFα, IL-6, and MCP-1) while increasing markers of mitochondrial biogenesis (e.g., PGC-1α, Tfam, and COX1) in the liver. We suggest that CCR2 inhibitor may ameliorate hepatic steatosis by reducing ER stress and inflammation in type 2 diabetes mellitus.

## Introduction

The liver is a vital organ for energy homeostasis and glucose metabolism. It absorbs and stores fatty acids from the blood and releases neutral fats into the blood as very-low-density lipoproteins when needed [1]. Accordingly, the liver is closely linked to metabolic disorders. Recently, many researches have focused on the close association between non-alcoholic fatty liver disease (NAFLD) and metabolic syndrome. Fatty liver may arise from type 2 diabetes or insulin resistance. Insulin resistance increases the expression of sterol regulatory element-binding protein (SREBP)-1c and fatty acid synthase (FasN) in the liver, elevating triglyceride (TG) accumulation [1, 2]. In addition, free fatty acids from adipose tissues migrate to the liver, which often cause fatty liver [3].

Accumulated TGs exacerbate insulin resistance in the liver. Furthermore, hepatic TG accumulation and cytokines released from adipose tissue damage the liver, causing inflammation and endoplasmic reticulum (ER) stress [4]. ER stress induces hepatic insulin resistance and mitochondrial dysfunction [5, 6]. ER stress also leads to C/EBP homologous protein (CHOP) and X-box binding protein 1 (XBP-1) activation. ER stress and mitochondrial dysfunction are associated with hepatic steatosis. Reduced mitochondrial biogenesis in the liver leads to the accumulation of liver fat [7].

Monocyte chemoattractant proteins (MCPs) and their receptors play pivotal roles in the development of inflammatory disorders, such as in hepatic steatosis, by recruiting immune cells to the area of inflammation [8]. MCP-1 belongs to the C-C chemokine family, which bind to C-C chemokine receptor 2 (CCR2) to initiate an inflammatory signal pathway [9]. The interaction between MCP-1 and CCR2 enhances the inflammation and ER stress [10]. CCR2 inhibitor potently competes against MCP-1 binding to CCR2 [11]. Macrophages in the liver contribute to inflammation through CCR2 binding with MCP-1 and CCR2 has been reported to enhance the accumulation of macrophages in steatohepatitis [12, 13]. Recent studies have reported that CCR2 inhibitor regulates fat and macrophage accumulation in adipose tissue, thereby improving NAFLD [14, 15]. In this study, we demonstrated that CCR2 inhibitor alleviates hepatic steatosis and elucidated how CCR2 inhibitor reduces hepatic steatosis.

## Materials and Methods

### 1. Animal models

Six-week-old C57BLKS/J *db/db* and *db/m* mice were purchased from Japan Shizuoka Laboratory Center (Shizuoka, Japan); *db/m* mice were used as controls in all experiments. The mice were divided into two groups: CCR2 inhibitor-treated mice and untreated controls. CCR2 inhibitor (RS102895) was purchased from Sigma-Aldrich (Sigma-Aldrich, St. Louis, MO, USA). Eight-week-old mice were fed either normal chow diet (NCD) or chow mixed with 2 mg/kg/day of RS102895 for 9 weeks. The amount of RS102895 added to NCD was adjusted according to the body weight of each mouse. Food and water intake, urine volume, body weight, and blood pressure were measured monthly. Blood glucose concentration was measured with SureStep (LifeScan, Milpitas, CA, USA). The animals were sacrificed 10 weeks after beginning treatment. All extracted tissues were immediately frozen in liquid nitrogen and stored at −80°C until analysis. All experiments were conducted in accordance with the National Institutes of Health guidelines and with the approval of the Yonsei University Institutional Animal Care and Use Committee (Wonju, Korea).

### 2. Cell culture

AML12 hepatocytes (ATCC, USA) were grown at 37°C in 5% CO₂ in Dulbecco’s modified Eagle’s medium/F12 (Gibco, NY, USA) containing 10% fetal bovine serum, 10 ml/L penicillin streptomycin (Invitrogen, Carlsbad, CA, USA). The medium was then replaced with DMEM/F12 containing 10% FBS and 100X Insulin-Transferrin-Selenium (ITS) (Gibco, NY, USA), and was changed every 2 days. Free fatty acids (palmitate mixture, Sigma-Aldrich) were dissolved in ethanol containing bovine serum albumin (BSA, 50 μM) and conjugated with BSA at a 10:1 molar ratio before use.

### 3. Hepatic triglycerides

Hepatic triglyceride (TG) content was assayed by saponification in ethanolic KOH, and glycerol content was measured with an FG0100 (Sigma-Aldrich) after neutralization with MgCl_2_. All tissue TG values were converted to glycerol content and corrected for liver weight.

### 4. Quantitative real-time PCR

Tissue RNA was extracted using TRIzol (Invitrogen), and total RNA (0.5 μg) was reverse-transcribed into cDNA according to the manufacturer’s instructions. For the quantitative, real-time, reverse transcriptase polymerase chain reaction (PCR) assays, the linearity of the amplification of SREBP1c, FASN, HSL, L-FABP, SCD1, mTOR, GLUT4, PGC-1α, COX1, Tfam, and GAPDH cDNAs was established in preliminary experiments. All signal mRNAs were normalized to GAPDH mRNA. cDNAs were amplified by real-time PCR in duplicate with a SYBR premix Ex Taq kit (Invitrogen) using Thermal Cycler Dice (Life Technologies, Carlsbad, CA, USA). Primers and optimal cycling conditions were as follows: mSREBP1c: sense 5’-GGA-GAC-ATC-GCA-AAC-AAG-CTG-A-3’, antisense 5’-AGA-CTG-CAG-GCC-AGA-TCC-A-3’; mFasN: sense 5’-AGC-ACT-GCC-TTC-GGT-TCA-GTC-3’, antisense 5’-AAG-AGC-TGT-GGA-GGC-CAC-TTG-3’; mHSL: sense 5’-TCC-TGG-AAC-TAA-GTG-GAC-GCA-AG-3’, antisense 5’-CAG-ACA-CAC-TCC-TGC-GCA-TAG-AC-3’; mL-FABP: sense 5’-AAG-TAC-CAA-TTG-CAG-AGC-CAG-GA -3’, antisense 5’-GGT-GAA-CTC-ATT-GCG-GAC-CA-3’; mSCD-1: sense 5’-TCG-CCC-CTA-CGA-CAA-GAA-CA-3’, antisense 5’-GTA-AGC-CAG-GCC-CA-3’; GAPDH: sense 5’-CTG-GAG-AAA-CCT-GCC-AAG-TA-3’, antisense 5’-AGT-GGG-AGT-TGC-TGT-TGA-AG-3’. All reactions were performed in the same manner: 95°C for 10 seconds, followed by 45 cycles of 95°C for 15 seconds and 60°C for 1 minute. The results were analyzed with real-time system AB 7900HT software (Life Technologies), and all values were normalized to the levels of GAPDH.

### 5. Western blot analysis

The liver was dissected and immediately frozen in liquid nitrogen. Three hundred milligrams of each liver was homogenized with 500 μL of lysis buffer (pH 7.4) supplemented with protease and phosphatase inhibitors. Lipids were removed by centrifugation at 10,000 *g* for 20 minutes. Fifty micrograms of total protein was subjected to Western blot analysis using a polyclonal antibody to phosphorylated Akt (Thr^308^), IRS-1 (Ser^307^), JNK (Thr^183^/Tyr^185^), or IKKβ (Ser^176/180^) or to non-phosphorylated Akt, IRS-1, JNK, IKKβ, CHOP, BiP (Cell Signaling, Beverly, MA, USA), CD68, IL-10, XBP-1, ATF4, SOD2, 4HNE (Santa Cruz Biotechnology, Santa Cruz, CA, USA), CD16, or β-actin (Abcam, Cambridge, UK). The band intensities were measured with an Image J Analyzer (Biocompare, San Francisco, CA, USA).

### 6. Measurement of cytokines by ELISA

The cytokines in tissue homogenates were assayed by enzyme-linked immunosorbent assays (ELISA). The assays were conducted using the OptEIA mouse TNF-α, murine MCP-1 set (BD Bioscience Pharmingen, Franklin Lakes, NJ, USA), and murine IL-6 antibody sets (R&D Systems, Minneapolis, MN, USA). Cytokine levels were quantified from standard curves using the BioTek curve-fitting program (BioTek, Winooski, VT, USA). Serum adiponectin levels were measured with a mouse adiponectin antibody set (R&D Systems).

### 7. Histological examination

Liver tissues were fixed overnight at room temperature in 4% formaldehyde and embedded in paraffin. Sections (8 μm thick) were stained with hematoxylin-eosin and were mounted on glass slides. The stained sections were viewed with an Axio-Star Plus light microscope (Carl Zeiss, Gottingen, Germany). Liver sections were stained with CD68 and XBP-1 antibodies (Santa Cruz).

### 8. Analysis of metabolic parameters

The mice were sacrificed after an 8-hour fast, and blood was collected through cardiac puncture. Serum triglyceride concentrations were determined using commercially available enzymatic assay kits (Asan Pharmacology, Seoul, Korea). Serum insulin levels were measured with an ultrasensitive mouse insulin ELISA kit (Shibayagi, Gunma, Japan).

### 9. Statistical analyses

All data are presented as mean ± SEM. Statistical analysis was performed using one-way analysis of variance (ANOVA) and Tukey’s test for multiple comparisons using SPSS version 17.0 (SPSS Inc., Chicago, IL, USA). The differences were considered to be statistically significant at *P* < 0.05.

## Results

### Changes in biochemical data from treated *db/db* mice

The fasting serum glucose (584.7 ± 31.1 *vs*. 401.5 ± 43.5 mg/dl) and insulin (15.3 ± 2.0 *vs*. 9.1±1.5 ng/ml) levels of *db/db* mice treated with CCR2 inhibitor were significantly lower than those of control *db/db* mice (Table 1). There were no differences among the *db/m* mice (Table 1). Serum TG (137.0 ± 16.3 *vs*. 100.2 ± 4.9 mg/dl) and ALT (74.6 ± 7.1 *vs*. 54.7 ± 4.6 IU/L) levels were significantly lower in treated *db/db* mice (Table 1). No significant differences in serum total cholesterol or AST levels were found between treated and control *db/db* mice (Table 1).

**Table 1:** Comparison of biochemical data among four groups.

	db/m	db/m+CCR2 i	db/db	db/db+CCR2 i
**Glucose (mg/dl)**	138.8±7.4	136.3±7.4	584.7±31.1*	401.5±43.5^#^
**Insulin (ng/ml)**	3.2±0.5	2.8±0.4	15.3±2.0*	9.1±1.5^#^
**TGs (mg/dl)**	94.4±6.1	81.5±2.8	137.0±16.3*	100.2±4.9^#^
**Total cholesterol (mg/dl)**	70.0±2.3	64.5±1.9	114.3±6.5*	105.0±3.9
**ALT (IU/L)**	13.0±0.7	6.6±0.6	74.6±7.1*	54.7±4.6^#^
**AST (IU/L)**	71.2±1.4	63.6±4.7	171.4±13.7*	167.1±9.4

*P < 0.05 compared with db/m mice.

#P < 0.05 compared to control db/db mice.

### CCR2 inhibitor prevents diabetes-induced hepatic steatosis in *db/db* mice

**Fig 1:**
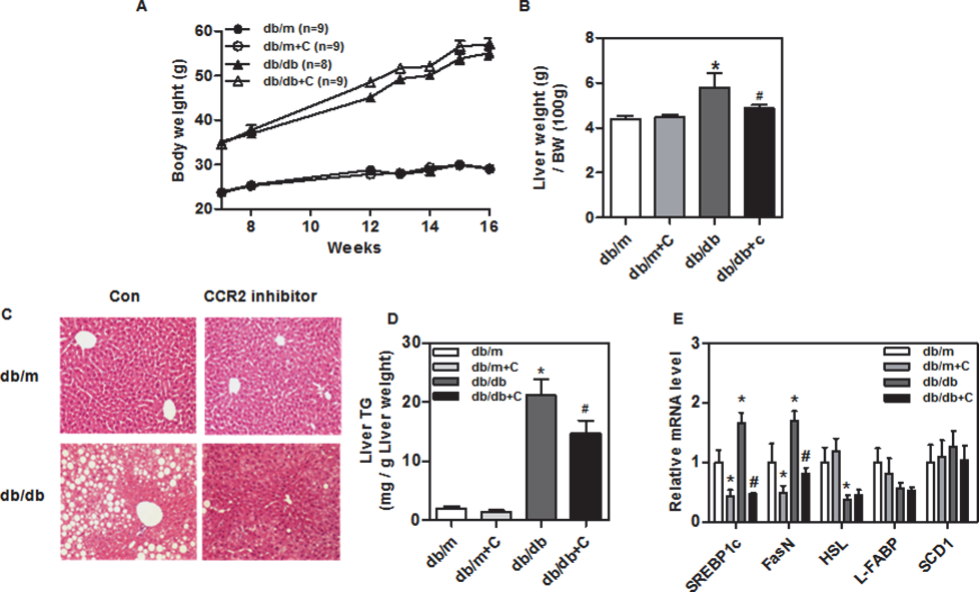
Changes in body weight, liver weight, TG content, and lipid synthesis genes in experimental animals. CCR2 inhibitor-treated and control *db/db* mice were fed an NCD for 9 weeks. (A) Body weight changes of mice and (B) liver weights of treated (n = 9) and control (n = 8) mice. (C) Histological analysis and (D) triglyceride (TG) content of liver. Sections were stained with hematoxylin and eosin. Results shown are means ± SEM. Original magnification is x200 (scale bar = 100 μm). (E) Levels of lipid synthesis gene (SREBP1c, FasN, HSL, L-FABP and SCD1) mRNAs in the livers of treated *db/db* mice. mRNA levels were estimated by real-time reverse transcriptase-PCR. Data shown are means ± SEM of 9 animals for each group. *P < 0.05 compared with the *db/m* mice. #P < 0.05 compared with control *db/db* mice.

Body weights did not differ between control and treated *db/db* mice (Fig. 1A). However, the average liver weight was significantly lower in treated *db/db* mice compared with control *db/db* mice (Fig. 1B). This difference was accompanied by smaller lipid droplets in the livers of treated *db/db* mice (Fig. 1C). The liver TG levels of treated *db/db* mice were significantly lower than those of control *db/db* mice (21.1 ± 2.7 *vs* 14.7 ± 2.2 mg/g liver weight) (Fig. 1D). The expression of fatty acid synthesis genes (e.g., SREBP-1c and FasN) was significantly reduced in the livers of treated *db/db* mice (Fig. 1E). This finding suggests that CCR2 inhibitor decreases lipid accumulation in *db/db* mice.

### Reduced inflammatory cell infiltration in the liver of treated *db/db* mice

**Fig 2:**
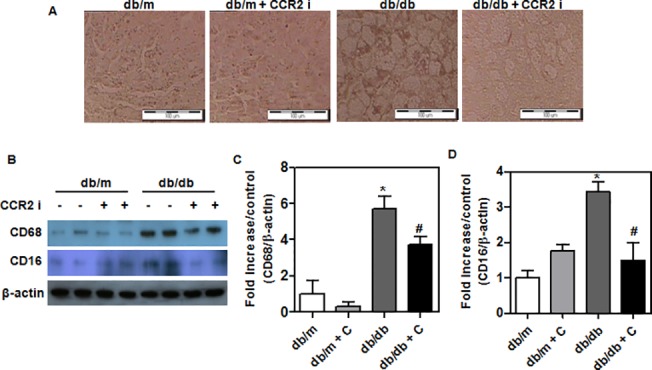
Changes of hepatic macrophages in experimental animals. (A) CD68 staining in the liver tissue sections by immunohistochemistry. Original magnification is x200 (scale bar = 100 um). (B) Western blots of (C) macrophage (CD68), and (D) M1 macrophage (CD16) in the liver of CCR2 inhibitor-treated (*n* = 9) and control (*n* = 8) mice. The band intensities were measured with an Image J Analyzer. Results shown are means ± SEM. *P < 0.05 compared with *db/m* mice. #P < 0.05 compared with control *db/db* mice.

To observe whether CCR2 inhibitor affects diabetes-induced hepatic inflammatory responses, we compared the infiltration of hepatic macrophages in the liver. Immunohistochemistry of the liver showed that CCR2 inhibitor reduced macrophage infiltration (CD68) (Fig. 2A). Western blot analysis showed reduced levels of a macrophage marker (CD68) in treated *db/db* mice (Fig. 2B and 2C), which was associated with reduced levels of an M1 macrophage marker (CD16) (Fig. 2D).

### Reduced inflammatory cytokine levels in *db/db* mice fed CCR2 inhibitor

**Fig 3:**
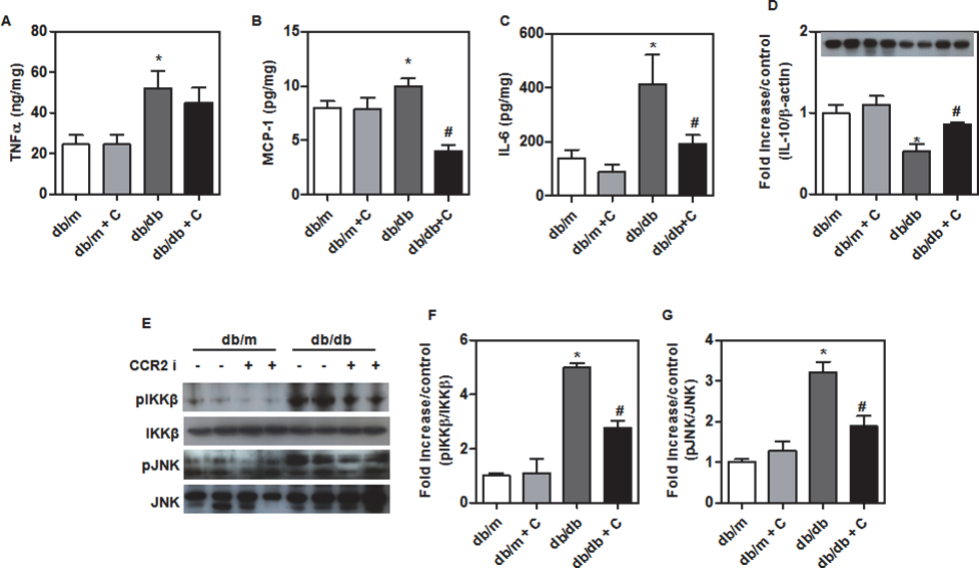
Hepatic inflammatory responses in experimental animals. (A-D) Concentrations of inflammatory proteins such as TNF-α, IL-6, MCP-1 and anti-inflammatory cytokine (IL-10) in the livers of CCR2 inhibitor-treated (*n* = 9) and control (*n* = 8) db/db mice. (A-C) Cytokine levels in homogenates were measured by enzyme-linked immunosorbent assay and normalized for protein content. (D) Western blots of anti-inflammatory cytokine (IL-10) in the livers of treated (*n* = 9) and control mice (*n* = 8). (E-G) Inflammatory signaling pathway markers IKKβ and JNK in the livers of treated (*n* = 9) and control mice (*n* = 8). The band intensities were measured with an Image J Analyzer. Results shown are means ± SEM. *P < 0.05 compared with *db/m* mice. #P < 0.05 compared with control *db/db* mice.

Treated *db/db* mice had lower levels of inflammatory cytokines (TNFα, MCP-1, and IL-6) in the liver compared with control *db/db* mice (Fig. 3A, 3B and 3C). Expression of a hepatic anti-inflammatory cytokine (IL-10) was higher in treated *db/db* mice (Fig. 3D). We also examined the effect of MCP-1 signaling on IKKβ and JNK and found that the reduction of p-IKKβ and p-JNK was larger in treated than control *db/db* mice (Fig. 3E, 3F and 3G).

### Activation of mitochondrial biogenesis, antioxidation and reduced ER stress in the livers of treated *db/db* mice

**Fig 4:**
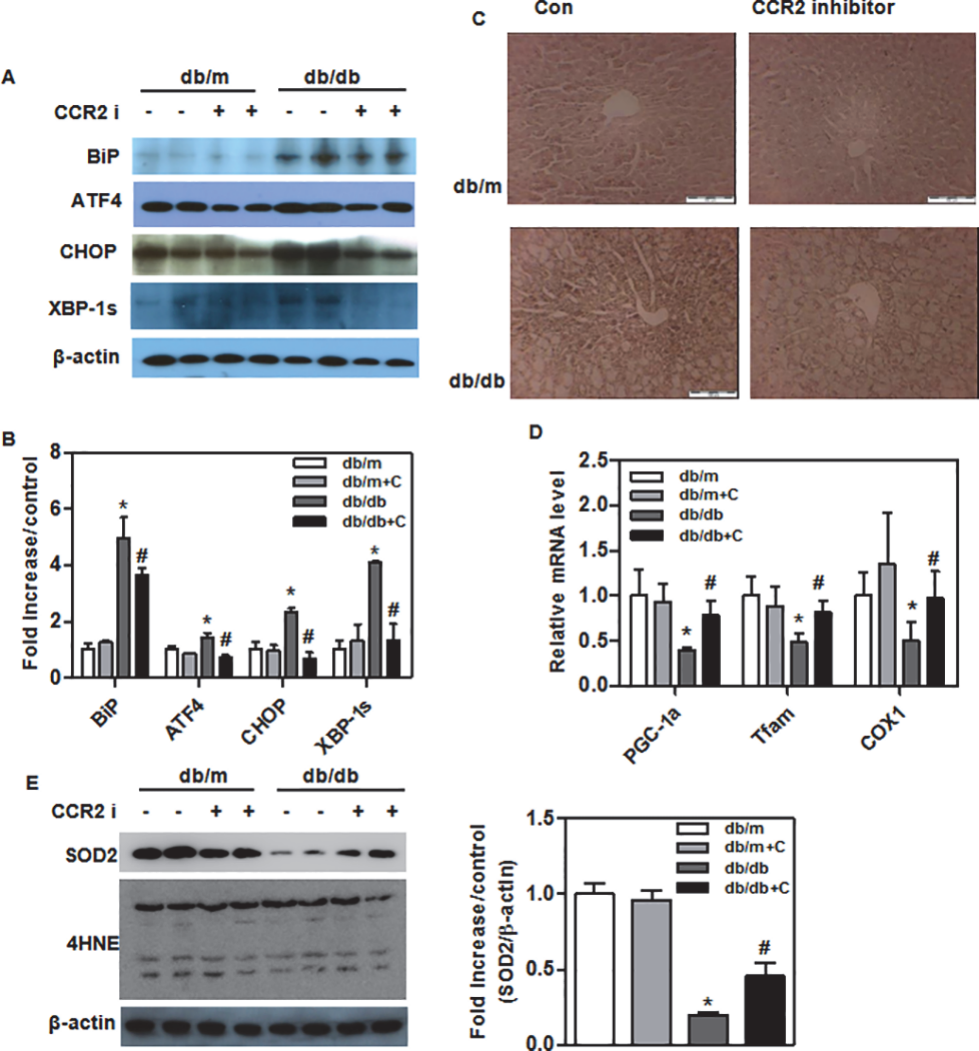
ER stress, oxidative stress proteins, and mitochondrial biogenesis gene levels in the livers of experimental animals. Liver tissue (300 μg) was homogenized with 500 μL of lysis buffer supplemented with protease and phosphatase inhibitors. Western blots showing levels of (A and B) ER stress proteins (BiP, ATF4, CHOP and XBP-1s) in livers of CCR2 inhibitor-treated (*n* = 9) and control (*n* = 8) *db/db* mice. (C) XBP-1 staining of liver sections by immunohistochemistry. Original magnification is x200 (scale bar = 100 um). (D) Levels of mitochondrial biogenesis genes (PGC1a, Tfam, and COX1) in liver from treated (*n* = 9) and control (*n* = 8) *db/db* mice. (E) Western blots showing levels of antioxidation (SOD2) and oxidative stress (4HNE) proteins in liver from treated (*n* = 9) and control (*n* = 9) mice. Band intensities were measured with an Image J Analyzer. Results shown are means ± SEM. *P < 0.05 compared with *db/m* mice. #P < 0.05 compared with control *db/db* mice.

The levels of BiP, ATF4, CHOP, and XBP-1 protein, which are ER stress markers, were significantly decreased in treated *db/db* mice (Fig. 4A and 4B). Immunohistochemistry of the liver showed reduced XBP-1 expression in treated *db/db* mice (Fig. 4C). We also examined the expression of genes related to mitochondrial biogenesis (e.g., PGC-1α, Tfam, and COX1), which were significantly elevated in CCR2 inhibitor-fed mice (Fig. 4D). SOD2 was also significantly elevated in the liver of treated *db/db* mice (Fig. 4E). The level of 4-HNE did not differ significantly between treated and control *db/db* mice (Fig. 4E). This observation suggests that CCR2 inhibitor decreases ER stress and increases mitochondrial biogenesis and antioxidation.

### Improvement in insulin signaling of treated *db/db* mice

**Fig 5:**
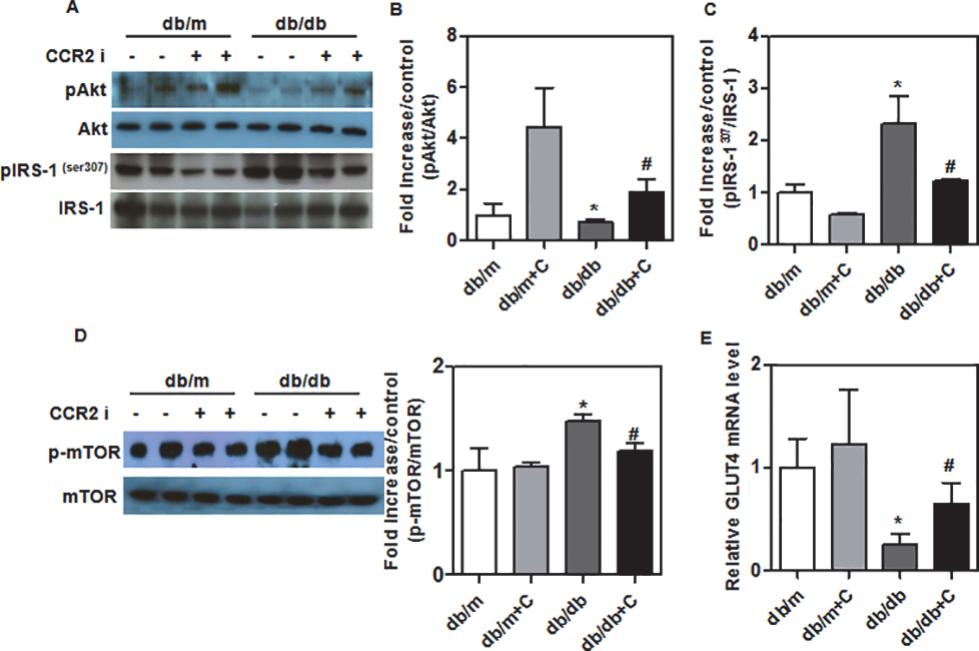
CCR2 inhibitor ameliorates insulin resistance in experimental mice. (A, B and C) Western blots of insulin signaling (pIRS-1 Ser^307^, IRS-1, pAkt and Akt) in liver from CCR2 inhibitor-treated (*n* = 9) and control (*n* = 8) *db/db* mice. (D) Western blots of mTOR in liver from CCR2 inhibitor-treated (*n* = 9) and control (*n* = 8) *db/db* mice. (E) GLUT4 mRNA in the livers of treated *db/db* mice. mRNA levels were evaluated by real-time reverse transcriptase-PCR. Bands intensities were measured with an Image J Analyzer. Results shown are means ± SEM (n = 8–9 mice per group). *P < 0.05 compared with *db/m* mice. #P < 0.05 compared with control *db/db* mice.

IRS-1 phosphorylation (Ser^307^) inhibits insulin signaling (16). mTOR and AKT play roles in GLUT4 translocation. Akt phosphorylation was significantly higher (Fig. 5B), and IRS-1 (Ser^307^) phosphorylation was significantly lower in the livers of treated *db/db* mice (Fig. 5C). mTOR phosphorylation was significantly lower in the livers of treated *db/db* mice (Fig. 5D). These changes were associated with increased expression of GLUT4 mRNA (Fig. 5E). These findings suggest that CCR2 inhibitor restores insulin signaling in *db/db* mice.

### CCR2 inhibitor prevents palmitate-induced hepatic steatosis in AML12 hepatocytes

**Fig 6:**
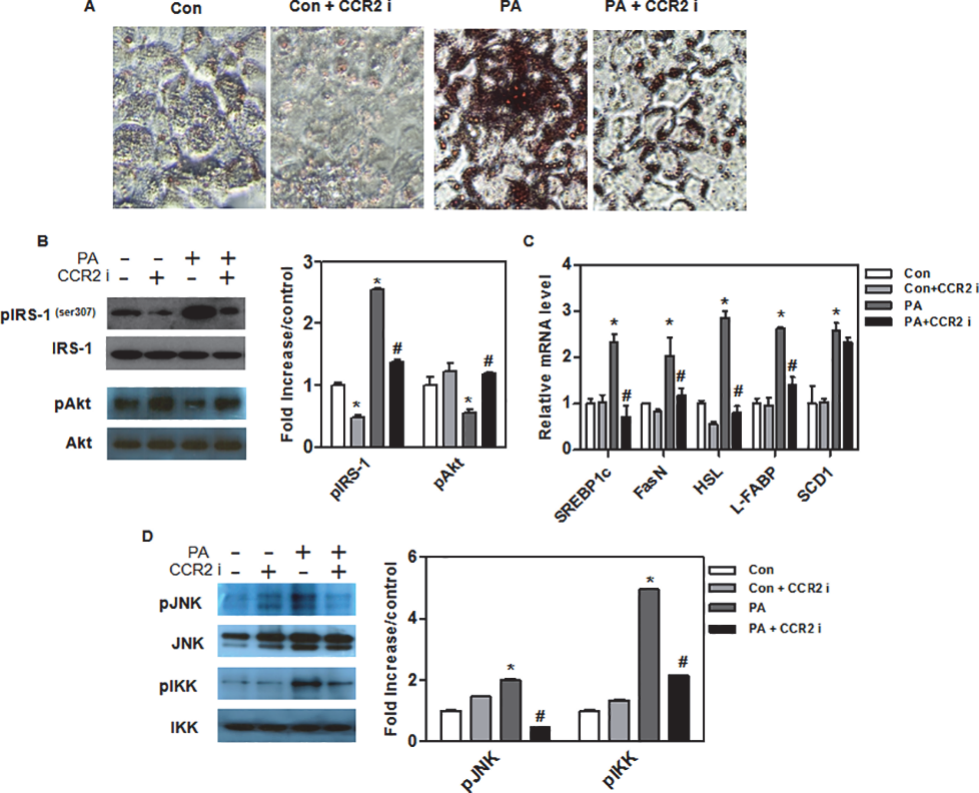
Upregulation of lipid synthesis on hepatocytes by palmitate. Hepatocytes (AML12) were treated with a fatty liver-related factor (palmitate 250 μM). Palmitate was prepared in ethanol containing bovine serum albumin (BSA, 50 μM). (A) AML12 cells were stained with Oil red O. Original magnification is x200 (scale bar = 100 μm). (B) Western blots of SREBP-1 and PGC-1a in AML12 cells. (C) mRNA levels of lipid synthesis genes (SREBP1c, FasN, HSL, L-FABP, and SCD1) in AML12 cells. (D) Western blots of inflammatory signaling pathway markers (IKKβ and JNK) in AML12 cells. Bands intensities were measured with an Image J Analyzer. Results shown are means ± SEM. *P < 0.05 compared with control. #P < 0.05 compared with palmitate-treated control.

Hepatocytes (AML12) were treated with palmitate (250 μM) for 24 hours. CCR2 inhibitor reduced lipid accumulation by 70% compared with palmitate alone (Fig. 6A). We treated AML12 hepatocytes with palmitate and assessed the effect of CCR2 inhibitor in the presence of rapamycin or Akt inhibitor. Interestingly, it seems that there was no synergistic effect in hepatic steatosis when cells were co-treated with CCR2 inhibitor and rapamycin (S1 Fig.). But, the treatment of cells with Akt inhibitor did not alter palmitate-induced steatosis (S1 Fig.). IRS-1 phosphorylation (Ser^307^) was significantly lower, and Akt phosphorylation was significantly higher in CCR2 inhibitor-treated AML12 hepatocytes (Fig. 6B). We examined the expression of genes for fatty acid synthesis and transport and found that they were significantly reduced in CCR2 inhibitor-treated AML12 hepatocytes (Fig. 6C). Western blot analysis showed lower levels of inflammatory signal markers (pIKK and pJNK) in CCR2 inhibitor-treated AML12 hepatocytes (Fig. 6D). This finding suggests that CCR2 inhibitor-treated AML12 hepatocytes decreased lipid accumulation compared with AML12 hepatocytes treated with palmitate alone.

### Decreased oxidative and ER stress in CCR2 inhibitor-treated AML12 hepatocytes

**Fig 7:**
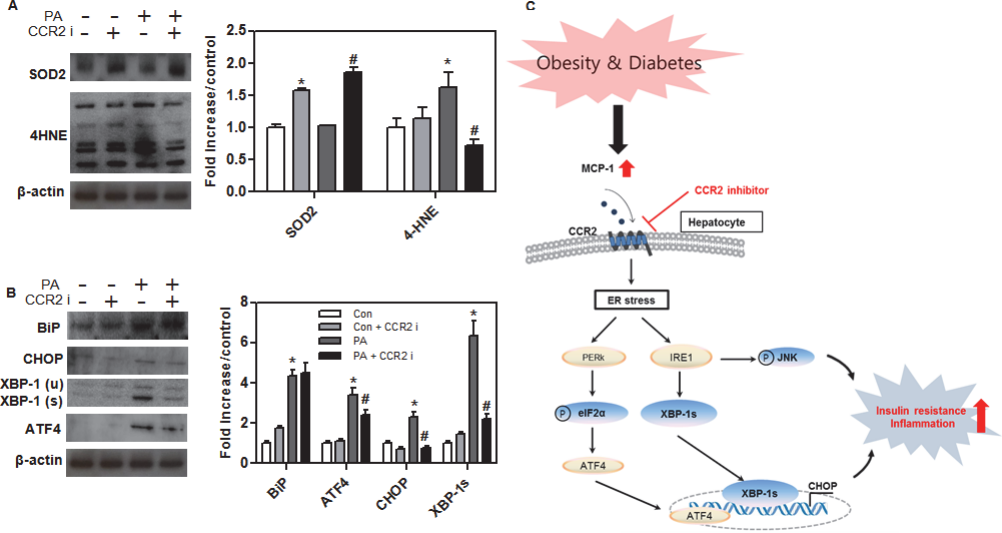
Hepatic inflammatory and ER stress responses in AML12 cells. Western blots of (A) oxidative stress markers (SOD2 and 4-HNE) and (B) ER stress proteins (BiP, ATF4, CHOP, and XBP-1s) in AML12 cells. Band intensities were measured with an Image J Analyzer. Results shown are means ± SEM. *P < 0.05 compared with control. #P < 0.05 compared with palmitate-treated control. (C) Schematic presentation for the effect of CCR2 inhibitor in hepatocyte.

Western blot analysis showed increased levels of an antioxidative marker (SOD-2) and decreased levels of an oxidative stress marker (4-HNE) in CCR2 inhibitor-treated AML12 hepatocytes (Fig. 7A). We confirmed that the protein levels of ATF4, CHOP, and XBP-1, which are markers of ER stress, were significantly decreased in CCR2 inhibitor-treated AML12 hepatocytes (Fig. 7B). We found that the treatment of cells with palmitate increased the expression of SREBP-1c and BiP, and which were significantly decreased by CCR2 inhibitor or rapamycin treatment (S2 Fig.). However, we could not observe any significant alteration by the cotreatment of CCR2 inhibitor and rapamycin (S2 Fig.). This observation suggests that CCR2 inhibitor decreases oxidative and ER stress in AML12 hepatocytes.

## Discussion

In this study, we investigated the protective effect of CCR2 inhibitor on hepatic steatosis in a diabetic mouse model. Hepatic steatosis is the most common chronic liver disease, and it is characterized by the accumulation of TGs in the liver [3]. The pathogenesis of NAFLD remains uncertain, but obesity, insulin resistance, ER stress, and inflammatory cytokines have been identified as the major factors involved. Given this complex collection of factors, two hypotheses have been suggested: one implicating the accumulation of liver fat and the other suggesting prompt steatosis progressing to non-alcoholic steatohepatitis (NASH) [17, 18]. In this study, the change in body weight did not differ between control *db/db* and treated *db/db* mice, but the liver weight decreased significantly in treated *db/db* mice. Moreover, hepatic TG levels decreased in treated *db/db* mice. SREBP-1c and FasN play important roles in controlling fatty acid synthesis in the liver [19, 20]. SREBP-1c acts as a transcription factor that regulates the genes controlling TG synthesis. SREBP-1c preferentially enhanced the transcription of genes required to synthesize fatty acids and cholesterol. This predilection could be due either to reduced TG synthesis or to increased hepatic fatty acid oxidation. CCR2 inhibitor reduced the mRNA levels of SREBP-1c and FasN *in vivo* and *in vitro*. This study suggests that CCR2 inhibitor plays an important role in regulating hepatic lipid homeostasis.

Recent studies have investigated the molecular mechanism of inflammatory signal activation and the role of Kupffer cells in hepatic insulin resistance [21]. Kupffer cells mediate inflammatory signals in the liver. Fatty liver resulting from a high-fat diet is associated with Kupffer cell activation [22]. The number of M1 macrophages was increased in fatty liver, initiating the inflammation associated with reduced insulin sensitivity [23, 24]. According to our study, decreased hepatic M1 macrophage infiltration in treated *db/db* mice may activate an anti-inflammatory response in the liver.

Kupffer cell activation releases pro-inflammatory cytokines and chemokines. Inflammatory cytokines (e.g., TNFα, MCP-1, IL-6) worsen insulin resistance and inflammatory signaling in mice [25]. TNFα, IL-6, MCP-1, and macrophage-secreted factors exert paracrine effects that activate inflammatory pathways within insulin target cells [26]. Several inflammatory signaling molecules, including IKKβ and JNK, also affect insulin sensitivity and glucose metabolism [27, 28]. Several other reports showed that ER stress could induce the inflammatory response through JNK phosphorylation [29–31]. In our study, the level of ER stress was significantly decreased in the CCR2 inhibitor-treated mice, suggesting that ER stress reduction in hepatocytes is possible to result in the reduced JNK activation, and which could ameliorate the hepatic inflammation. We found that administering CCR2 inhibitor downregulated pro-inflammatory cytokines (TNFα, MCP-1 and IL-6) and increased an anti-inflammatory cytokine (IL-10) in the liver. Treated *db/db* mice had improved glucose tolerance in the liver, which resulted from decreased recruitment of hepatic M1 macrophages, leading to reduced cytokine levels. These findings imply that CCR2 inhibitor reduces inflammatory responses in fatty liver by reducing the recruitment of specific macrophages, thereby ameliorating hepatic ER stress.

Chronic ER stress affects risk factors associated with dyslipidemia and diabetes [32]. Recent studies have demonstrated that FFA-induced ER stress activates the unfolded protein response (UPR), leading to increased expression of SREBP-1c and genes responsible for fatty acid synthesis [20]. The protein levels of ER stress markers, including BiP and CHOP, are increased in human subjects with metabolic syndrome [5]. The UPR response to high glucose seems to be associated with increased expression of inflammatory cytokines via IRE-1-JNK pathway [33, 34]. Down-regulating CHOP activity can ameliorate the detrimental effects of metabolic disorders by attenuating inflammatory cytokine release. ER stress also enhances insulin resistance induced by mitochondrial dysfunction [35]. In this study, CCR2 inhibitor reduced the protein levels of ER stress markers (BiP, ATF4, CHOP, and XBP-1) *in vivo* and *in vitro*. The expression of mitochondrial biogenesis genes (PGC-1α, Tfam, and COX1) increased significantly in the livers of treated *db/db* mice compared with control *db/db* mice. The decreased ER stress markers and increased mitochondrial biogenesis markers in the liver might indicate a pathway by which hepatic steatosis improved in treated *db/db* mice.

Recent reports have shown that accumulated hepatic TGs result in insulin resistance by inhibiting insulin receptor signaling. Insulin resistance is a crucial pathological factor in the progression and development of NAFLD [36]. Our previous study showed that insulin resistance was significantly improved in the treated *db/db* mice compared with control *db/db* mice [37]. In this study, we found that CCR2 inhibitor did not influence fasting blood TG, glucose, or insulin levels in *db/m* mice. However, CCR2 inhibitor did significantly reduce those parameters in *db/db* mice. Insulin is a key hormone regulating carbohydrate and lipid metabolism. Phosphorylated Akt induces mTOR and GLUT4 in the mitochondria [38]. Serine phosphorylation of IRS-1 has been shown to inhibit the insulin receptor signaling pathway because it inhibits insulin-stimulated tyrosine phosphorylation of IRS-1 [16]. We found that CCR2 inhibitor significantly increased Akt phosphorylation, along with decreasing serine phosphorylation of IRS-1 in vivo and in vitro. Interestingly, although it seems that the treatment of cells with Akt inhibitor did not alter palmitate-induced steatosis and ER stress responses, Akt inhibitor partly reversed the effect of CCR2 inhibitor on steatosis and SREBP1c expression, suggesting that the effect of CCR2 blockade, including reduced steatosis and ER stress, might be partially through increased Akt signaling. Cotreatment of CCR2 inhibitor and rapamycin showed additive effect on the IRS1 phosphorylation. However, there was no significant change in IRS1 phosphorylation by Akt inhibition in palmiate-treated AML12 hepatocytes.

No other studies so far have described a detailed mechanism involving CCR2 inhibitor and NAFLD, except for its inhibition of adipose tissue-induced hepatic steatosis. This study is the first to describe the mechanism of CCR2 inhibitor in hepatic steatosis, and supports the involvement cross talk between MCP-1 and CCR2, which could activate inflammatory responses and blunt insulin signaling in the fatty liver. Therefore, CCR2 inhibitor may be a useful therapeutic agent in ameliorating fatty liver in type 2 diabetes.

Despites, there are still several limitations in our study. First, we could not elucidate the exact mechanism of inflammatory signaling regulation by CCR2 inhibitor. Further study should be done to reveal a clear relationship of inflammation through the treatment of NF-κB or MAPK inhibitor. Secondly, we used AML12 mouse hepatocytes cell line in vitro. Primary hepatocyte is more close to the character of mouse liver. Although, AML12 hepatocytes cell line is also normal hepatocytes, it is different with primary hepatocytes. Finally, we could not confirm how CCR2 inhibitor implicated CCR2 binding with MCP-1 and only suggested that it competed against MCP-1 binding to CCR2.

## Supporting Information

S1 Fig Lipid accumulation on AML12 hepatocytes by palmitate. Hepatocytes (AML12) were treated with a fatty liver-related factor (palmitate 250 μM). Palmitate was prepared in ethanol containing bovine serum albumin (BSA, 50 μM). AML12 cells were stained with Oil red O. Original magnification is x200 (scale bar = 100 μm).
(TIF)

S2 Fig Hepatic inflammatory and ER stress responses in AML12 cells. Western blots of BiP, SREBP-1, p-IRS1 and IRS1 in AML12 cells. Bands intensities were measured with an Image J Analyzer. Results shown are means ± SEM. *P < 0.05 compared with control. #P < 0.05 compared with palmitate-treated control.
(TIF)
