# Supplementary figures and images for: C-C Chemokine Receptor 2 Inhibitor Ameliorates Hepatic Steatosis by Improving ER Stress and Inflammation in a Type 2 Diabetic Mouse Model
Source: PLoS One. 2015 Mar 27;10(3):e0120711. doi: 10.1371/journal.pone.0120711 (PMC4376739; doi:10.1371/journal.pone.0120711)

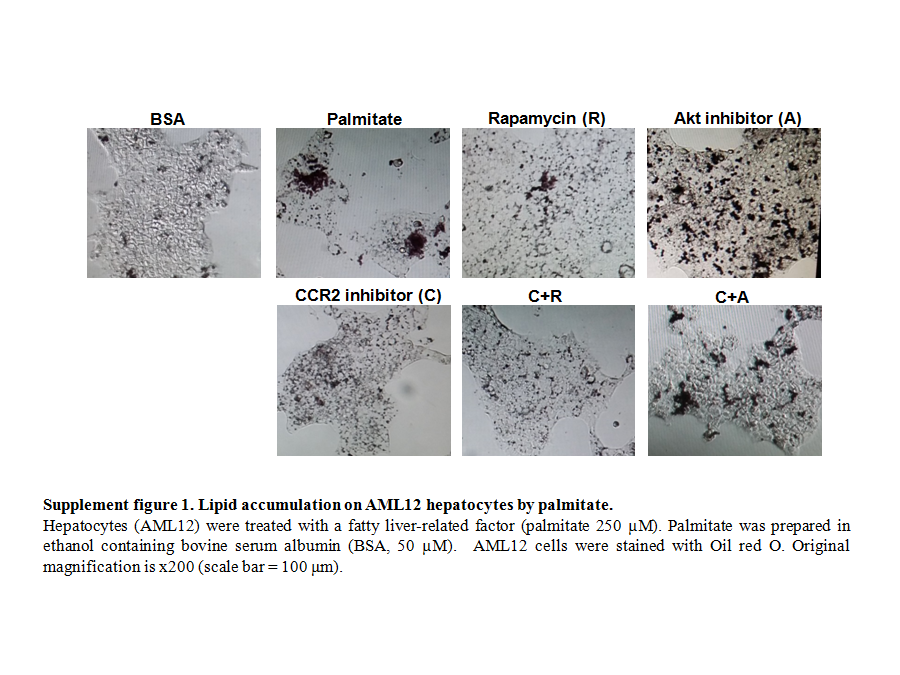

Supplement: S1 Fig — Hepatocytes (AML12) were treated with a fatty liver-related factor (palmitate 250 μM). Palmitate was prepared in ethanol containing bovine serum albumin (BSA, 50 μM). AML12 cells were stained with Oil red O. Original magnification is x200 (scale bar = 100 μm). (TIF) [file pone.0120711.s001.TIF]

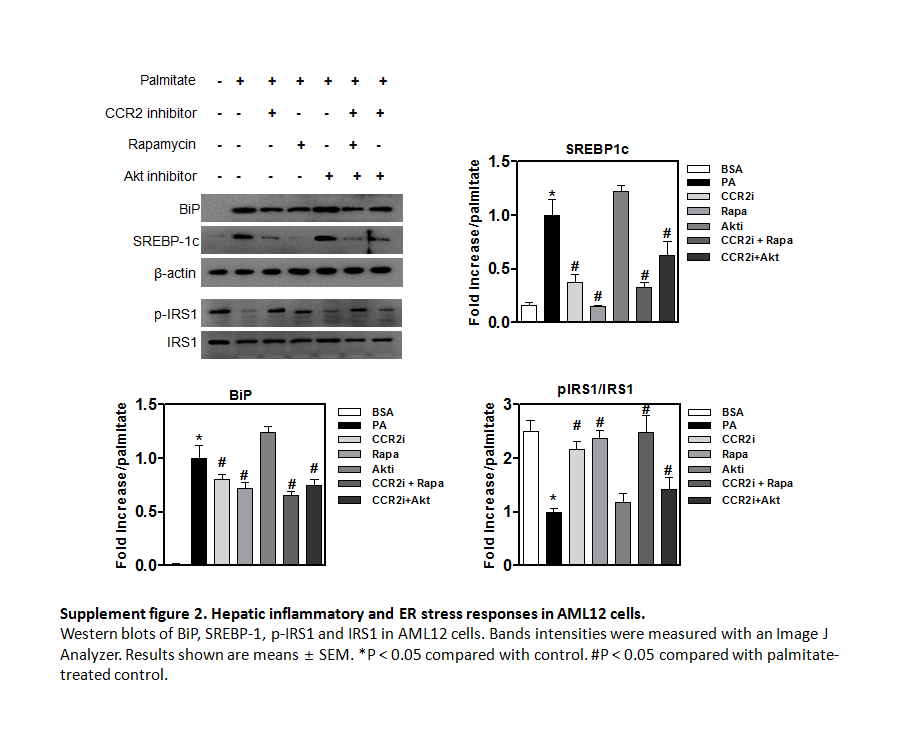

Supplement: S2 Fig — Western blots of BiP, SREBP-1, p-IRS1 and IRS1 in AML12 cells. Bands intensities were measured with an Image J Analyzer. Results shown are means ± SEM. *P < 0.05 compared with control. #P < 0.05 compared with palmitate-treated control. (TIF) [file pone.0120711.s002.TIF]
